# The Effect of Copper-Free, Low-Metal Molybdenum Disulfide Friction Pads on Carbon–Ceramic Friction Pads

**DOI:** 10.3390/ma19214567

**Published:** 2026-10-29

**Authors:** Wenmin Kuang, Xiang Xiong, Xin Chu, Hua Wang, Yin Jian

**Affiliations:** 1 State Key Laboratory for Powder Metallurgy, Central South University, Changsha 410083, China; 2 KPC High-Tech Co., Ltd., Changsha 413000, China

**Keywords:** low metal friction pads, carbon-ceramic friction pads, molybdenum disulfide (MoS_2_)

## Abstract

This study systematically investigated three types of low-metal friction pads with different molybdenum disulfide (MoS_2_) contents and analyzed their physicochemical properties and friction and wear performance. Test results show that the A2 specimen, containing 8% MoS_2_, exhibited the best overall physicochemical properties, with shear strength and compressive strength reaching 6.69 ± 0.52 MPa and 61.77 ± 1.56 MPa, respectively, while also demonstrating the best hydrophobicity. Under braking conditions, the A2 specimen exhibited the lowest coefficient of friction (0.41) and a significantly reduced wear rate; compared to the A0 specimen without MoS_2_ addition, wear was reduced by 50%. In summary, MoS_2_ shows great application potential and is expected to become one of the core solid lubricants for automotive brake pads.

## 1. Introduction

### 1.1. MoS_2_

Solid lubricants have been in use for nearly five decades and are commonly employed for the lubrication and protection of mechanical components under low-pressure, high-temperature conditions; they exhibit exceptional adaptability in the field of friction materials [1]. Currently, solid lubricants used in high-temperature lubrication can be classified into five major categories based on their lubrication properties and mechanisms: soft metals, metal oxides, metal fluorides, layered-structure lubricants, and polymers [2,3,4]. Typical solid lubricants such as graphene, molybdenum disulfide (MoS_2_), and hexagonal boron nitride (hBN) all possess unique two-dimensional layered crystal structures, enabling them to exhibit extremely low coefficients of friction. They are highly suitable for various tribological applications, including wear-resistant protective coatings and solid-state lubricants [5,6,7,8]. Among the solid lubricants currently in use, molybdenum disulfide (MoS_2_) holds particular significance. As a layered solid material composed of single-atom-thick planes that can slide easily against one another, the use of MoS_2_ as a solid lubricant in modern technology dates back to the last century [9,10]. MoS_2_ possesses a typical quasi-two-dimensional layered crystal structure [11]. The intralayer bonding is dominated by strong covalent interactions within the S-Mo-S sheets, whereas adjacent layers are held together by weak van der Waals forces with an extremely low shear strength of merely 1–2 MPa. This unique structural characteristic enables the formation of a slippery transfer film at the coating–counterpart interface, thereby achieving facile shear and low-friction behavior under diverse service environments [12]. Under frictional contact, MoS_2_ grains undergo friction-induced reorientation and preferentially align along the sliding direction with their (0001) basal planes exposed. Owing to their higher surface energy, these basal planes exhibit superior oxidation resistance and hydrophobicity compared with edge planes, thus imparting enhanced structural stability under applied shear stress [13]. However, when exposed to elevated temperatures above 400 °C under simultaneous shear loading, the (0001) basal slip systems of MoS_2_ undergo severe thermally activated oxidation. This oxidative degradation leads to elevated interfacial adhesion, reduced cohesive strength within the bulk coating, pore nucleation, and the generation of coarse wear debris [14]. The formed debris is subsequently extruded from the transfer film, resulting in direct asperity contact between the counterpart and substrate and a drastic degradation in lubrication efficiency. These drawbacks severely limit the high-temperature applicability of MoS_2_-based lubricating systems. Consequently, extensive investigations have been conducted over the past few decades to enhance the tribological properties of MoS_2_-based materials across a broad temperature spectrum, particularly under high-temperature conditions.

In view of the lubrication failure mechanism, the core strategy to improve the high-temperature lubricating performance and service life of MoS_2_-based coatings is to fabricate composite coatings by introducing additives. The synergistic effect among coating components is exploited to optimize surface properties and suppress water- and oxygen-induced oxidation. Hu et al. [15] fabricated multilayer MoS_2_/Sb_2_O_3_ composite films, which exhibit a much longer wear life than pure MoS_2_ films in air with a relative humidity of 40%. This improvement is attributed to the fact that the composite structure can effectively preserve the fresh MoS_2_ lubricating phase continuously supplied at the frictional interface and restrain crack propagation perpendicular to the interface. Gardos [16] found that the incorporation of graphite additives can effectively prolong the wear life of polished or bonded MoS_2_ coatings. The underlying mechanism is that the smoother basal planes of oxidized graphite facilitate the maintenance of interlayer shearing and delay the interlocking failure between coating layers. Kong et al. [17] reported a ZrO_2_-MoS_2_-CaF_2_ self-lubricating composite. Benefiting from the synergistic lubrication between MoS_2_ and CaF_2_, stable lubrication can be realized for this material at an elevated temperature of 1000 °C under ambient air and a low load of 10 N. Furthermore, NiAl-matrix self-lubricating composites with MoS_2_ and Ti_3_SiC_2_ as lubricating phases show outstanding self-lubricating properties over a broad temperature range from room temperature to 800 °C under pure Ar atmosphere at 10 N, which also originates from the synergistic interaction of the two phases. At present, the favorable low-friction performance of MoS_2_-based composite coatings is mostly achieved under high vacuum, inert atmosphere (dry Ar), and moderate-to-low temperature conditions (<400 °C). To the best of our knowledge, high-efficiency lubrication under the coupled conditions of open atmosphere and high temperature has rarely been reported.

### 1.2. Carbon/Silicon Carbide (C/C-SiC) Ceramic Brake Disks

Currently, the friction pads used in automotive braking systems primarily include gray cast iron (GCI) brake disks, surface-modified cast iron brake disks, aluminum and aluminum alloy brake disks, and high-performance (C/C-SiC) ceramic brake disks [18]. Gray cast iron brake disks are currently the most widely used brake disk material; they not only offer high strength and excellent wear resistance but also possess good vibration-damping properties and are inexpensive to manufacture. Aluminum and aluminum-based composite brake disks, on the other hand, offer advantages such as low density, high specific strength, high hardness, a low coefficient of thermal expansion, and outstanding wear resistance. C/SiC ceramic–composites have attracted considerable attention owing to their excellent tribological properties. Incorporating carbon fibers into the SiC ceramic matrix can markedly improve the tribological performance and toughness of composites, rendering them ideal candidates for high-performance friction materials. Studies have demonstrated that the tribological behavior of C/SiC composites is strongly affected by friction operating conditions including sliding velocity, normal load and ambient temperature. Third-body abrasive wear occurs during material wear processes: under low loads, third-body abrasive particles are primarily carbon-fiber fragments, whereas under high loads, these particles consist of both matrix particles and fiber fragments. Debris generated during friction and wear can form a stable protective film at the frictional interface, thereby improving lubrication and wear-resistance properties [19]. Carbon–ceramic brake materials maintain stable wear resistance and friction coefficient under both dry and humid environments [20]. Disk-on-disk counterpart wear tests reveal that braking velocity exerts a prominent effect on the temperature rise in C/SiC materials, while braking pressure has a relatively minor influence [21]. Moreover, the oxide reaction layer formed on C/SiC surfaces can effectively optimize the interfacial frictional state; introducing graphite phase into needled C/SiC composites can further enhance their wear resistance [22]. C/C-SiC brake disks have a density of only 1.8–2.2 g/cm^3^, while offering excellent oxidation resistance and wear resistance. Their wear rate per braking cycle is 0.5–2 × 10^−3^ mm, with a flexural strength of 300–450 MPa and a fracture toughness exceeding 10 MPa·m^1/2^. These brake disks can rapidly deliver maximum braking force during the initial braking phase without the need for additional braking assist mechanisms. Compared to traditional braking systems, they offer a faster braking response and shorter braking travel. Today, C/C-SiC brake disks have become the ideal choice of material for lightweighting automotive braking systems [23,24,25].

Li et al. [26] compared the friction and wear properties of powder–metallurgy, C/C and C/C-SiC friction pads mated with C/C-SiC brake rings. The results show that the coefficient of friction (COF) of the C/C-SiC self-mated pair is higher than that of the other two pairs, yet its COF stability is obviously inferior to those of the C/C pair and powder–metallurgy pair. Moreover, the wear rate of the C/C-SiC self-mated pair is lower than that of the powder–metallurgy counterpart only at a braking speed of 60 m/s. Krenkel et al. [27] investigated the tribological performance of short-fiber-reinforced C/C-SiC brake pads against commercial C/C-SiC brake disks, in comparison with low-metallic friction pads. It turns out that both the wear rate and COF of C/C-SiC brake pads are one order of magnitude higher than those of conventional low-metallic brake pads.

### 1.3. Copper-Free Low-Metallic Brake Pads

The primary reason for selecting copper-free friction materials for the braking system is to manage the ecological and environmental risks posed by copper emissions, in line with various environmental regulations. The U.S. Environmental Protection Agency (EPA) has issued relevant standards stipulating that the copper content in mass-produced brake pads must be limited to 5% or less for 2021 and beyond, with the limit further tightened to 0.5% by 2025. At the same time, the use of harmful metals and mineral components—such as antimony, asbestos, chromium, lead, mercury, and cadmium—is also strictly restricted. The Euro 7 regulation enacted by the European Union further raises emission control standards for automotive exhaust and brake pollutants, aiming to reduce pollutant emissions and improve air quality [28,29]. Recent studies have confirmed that copper residues released by brake pads and brake linings during service pose severe threats to the ecological environment, especially aquatic organisms such as salmon. Copper can disrupt the aquatic food chain and cause massive mortality in fish and other aquatic species. Wear debris from brake pads constitutes a major source of copper particles in the environment. After depositing on the ground, these copper particles enter streams, rivers and seawater, thereby exerting adverse toxic effects on aquatic organisms [30].

Due to increasing awareness of the health risks associated with asbestos, non-asbestos organic (NAO) fiber-reinforced low-metal friction composite materials are increasingly being used for automotive brake disk pads, shoes, linings, blocks, and clutch surfaces. Asbestos fibers are gradually being banned because they are prone to producing carcinogens at high temperatures and are harmful to human health [30]. Currently, commercial brake pads are mainly divided into three categories: low-metallic (LM), semi-metallic (SM), and non-asbestos organic (NAO) brake pads [31].

LM brake pads exhibit a high coefficient of friction and excellent braking performance under high-temperature conditions but suffer from a relatively high wear rate. SM brake pads deliver favorable braking properties and low wear resistance, yet they are prone to generating severe braking noise during operation. NAO brake pads feature low wear rates and outstanding noise reduction performance, whereas their braking capacity degrades significantly at high temperatures, leading to potential braking failure. Benefiting from superior comprehensive performance, NAO brake pads have become the most widely used type in the current market, accounting for nearly 80% of the market share. As one of the most crucial functional components in brake pads, copper can improve thermal conductivity, promote the formation of a stable friction layer at the friction interface, and reduce material wear. However, due to the adverse effects of copper on human health and the environment, its application in braking materials has gradually been restricted. During the braking process, copper-containing braking materials produce fine wear particles, which are washed into waterways by rainwater from the road surface and pose a threat to aquatic organisms [32]. The copper-containing wear particles can also harm aquatic organisms, for example, through interfering with the normal spawning process of salmon [33]. In terms of human health, the released copper particles can damage the tight junctions between the epithelial cells of the human lungs, and this mechanism involves the enhancement of oxidative stress and inflammatory responses. Previous studies have shown that copper-rich braking wear particles are correlated with the expression level of the tight junction protein occludin. In addition, human lung epithelial cells cultured using the gas–liquid interface (ALI) have been widely used to assess the toxic effects of exposure to airborne particulates. To address these issues, researchers have adopted alternative fillers and reinforcing materials, such as steel fibers, graphite, flax fibers, rock wool fibers, natural fibers, and barite, to develop environmentally friendly copper-free braking composite materials [34].

Currently, there is very little literature on the wear behavior of carbon–ceramic brake disks in the presence of molybdenum disulfide-based composites, and the effect of molybdenum disulfide concentration on friction properties warrants attention. Therefore, this paper comprehensively analyzes the importance of MoS_2_ as a solid lubricant and conducts an in-depth study of key aspects such as tribological properties.

## 2. Experimental Methods

### 2.1. Materials and Methods

**Figure 1:**
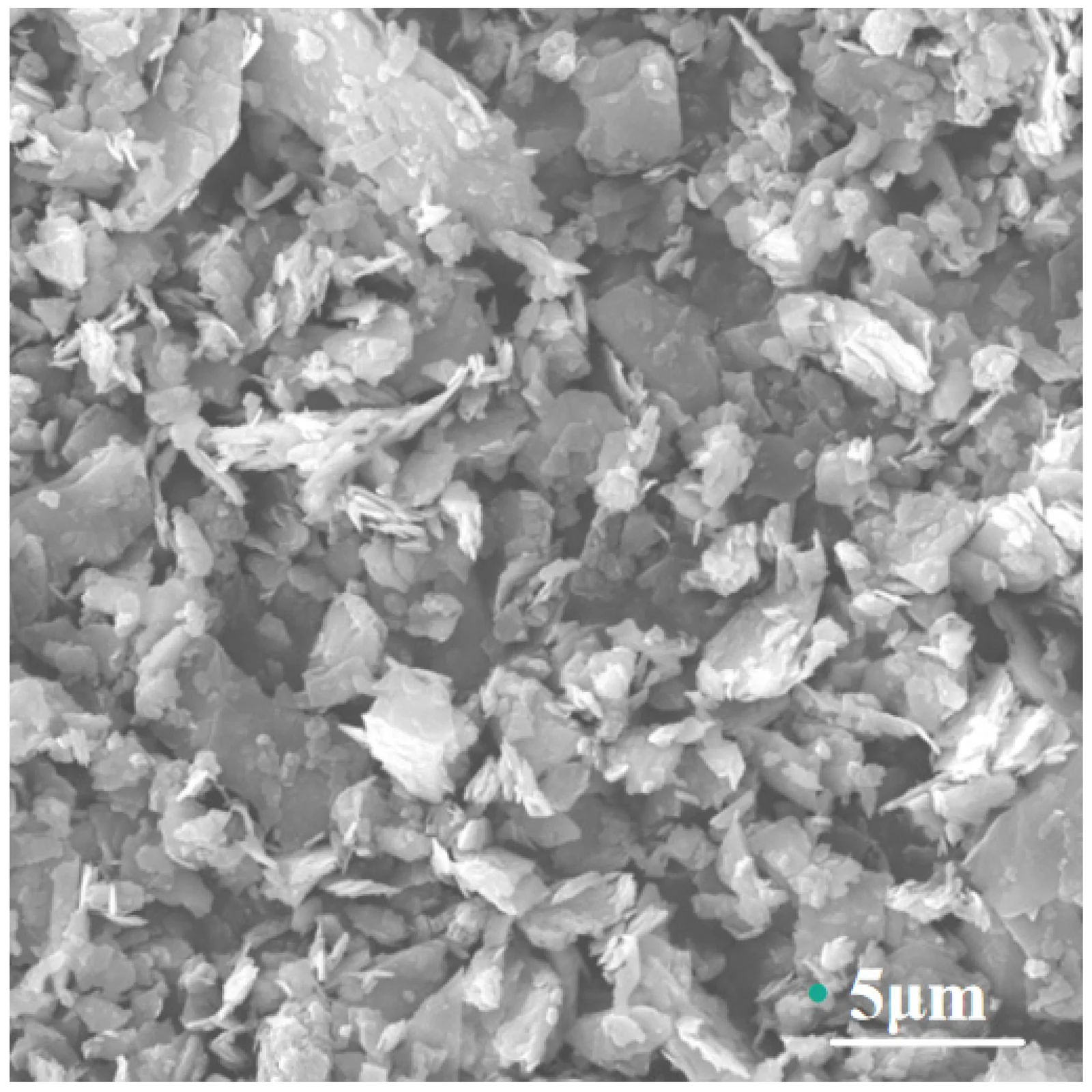
SEM image of MoS_2_.

**Figure 2:**
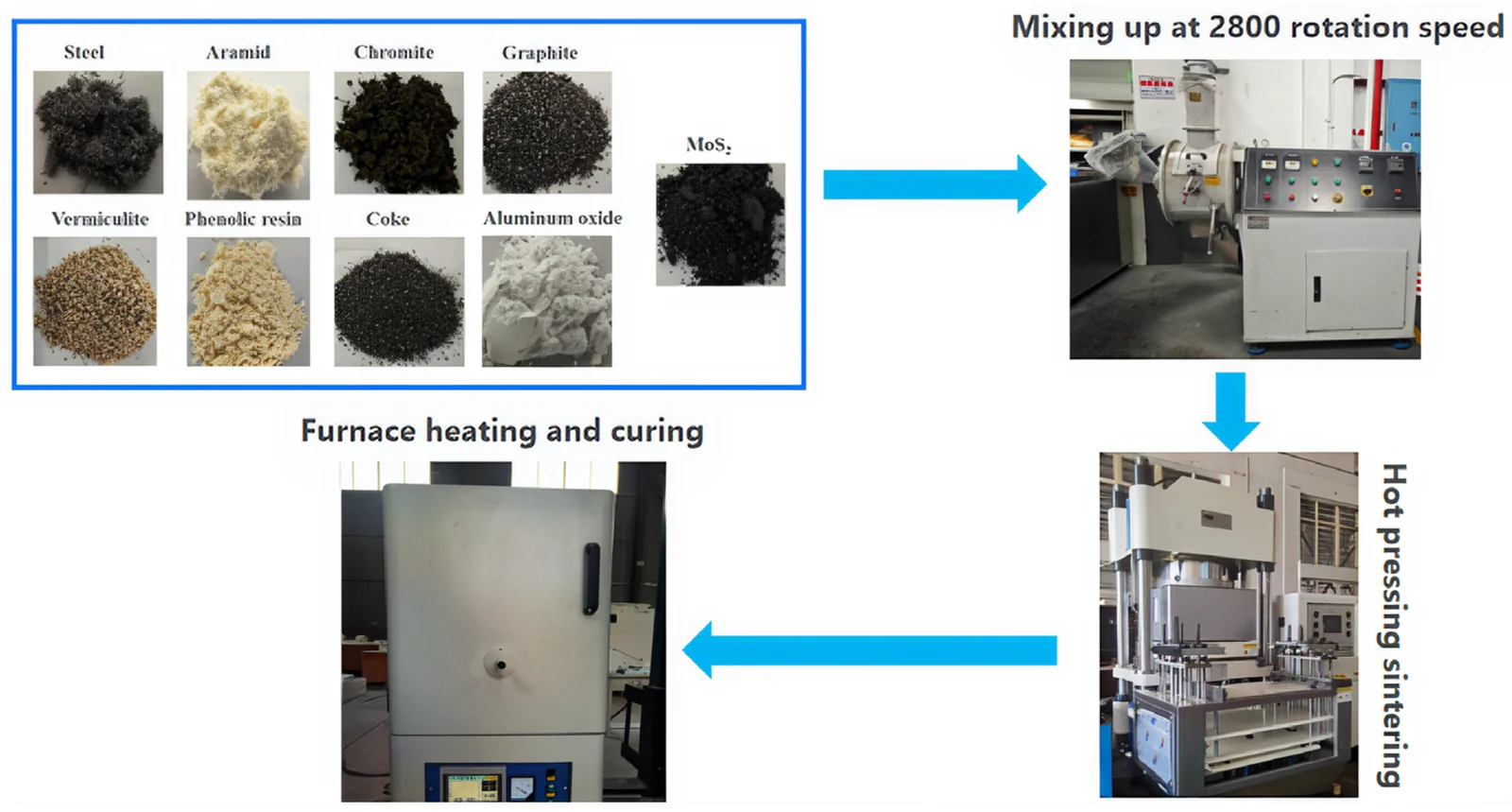
Flowchart of the preparation process for copper-free friction pads based on low-metal (LM) molybdenum disulfide.

In this study, low-metal, copper-free friction pads with molybdenum disulfide (MoS_2_) doping levels of 0%, 4%, and 8% by mass were prepared and labeled A0, A1, and A2, respectively. During the experiments, to ensure mass conservation in the sample formulations, the MoS_2_ content was adjusted sequentially, from 0% to 4% and 8%, while the proportions of the remaining components were kept constant. The mixed powders were then placed in molds (model WVA: 25887/25888) for hot-pressing. The raw material ratio parameters of the low-metal, copper-free friction sheets at different MoS_2_ loading levels are shown in Table 1. Figure 1 illustrates the microstructure of MoS_2_. Lubricants produce different lubricating effects depending on their structural shape and particle size. As shown in Figure 1, the MoS_2_ used in this study has a flake-like structure with a particle size of approximately 5 μm. Compared to ordinary 40 μm graphite flakes, the fine flake-like MoS_2_ can effectively form a third-body protective film during automotive braking, thereby reducing wear on the brake rotor and friction pads. The formulation used in the experiments was optimized through multiple trials and exhibits excellent overall performance. The preparation friction pads mainly includes the following steps: first, weigh and mix the raw materials according to the predetermined formula, then feed the prepared materials into a mixer for blending and stirring to produce the friction pads prepreg. The prepreg undergoes hot-pressing, curing, and then ablation, ultimately yielding the finished friction pads. During the testing process, a Mettler electronic balance with an accuracy of 0.001 g was used to weigh the raw materials. Then, the material is placed in a 50 L plow-type laboratory mixer for uniform mixing. After mixing, the composite powder is placed into the Xiaomi MS11-P mold for molding via hot-pressing. The molding pressure is set to 40 N/mm^2^, and the specific temperatures of the upper mold, core mold, and mold are set to 155 °C, 150 °C, and 150 °C, respectively. The hot-pressing process consists of three pressure cycles, each cycle including a 10 s pressure holding and compaction stage, followed by a 10 s exhaust and pressure-relief stage. After the cycle is completed, a final pressure holding phase of 150 s is carried out to solidify. The sample formed by hot-pressing is cured at a constant temperature of 200 °C for 12 h, and finally subjected to 3 min of ablation at 400 °C to form the final finished friction plate. Finally, the friction pad surfaces were ground and polished to prepare them for subsequent performance testing. The complete preparation process for the MoS_2_-containing, low-metal, copper-free friction pads is shown in Figure 2. The approximate compositional makeup of the three types of MoS_2_-containing, low-metal friction materials is presented in Table 1. Table 2 lists the test conditions for SAE-J2522.

### 2.2. Tribological Testing

**Figure 3:**
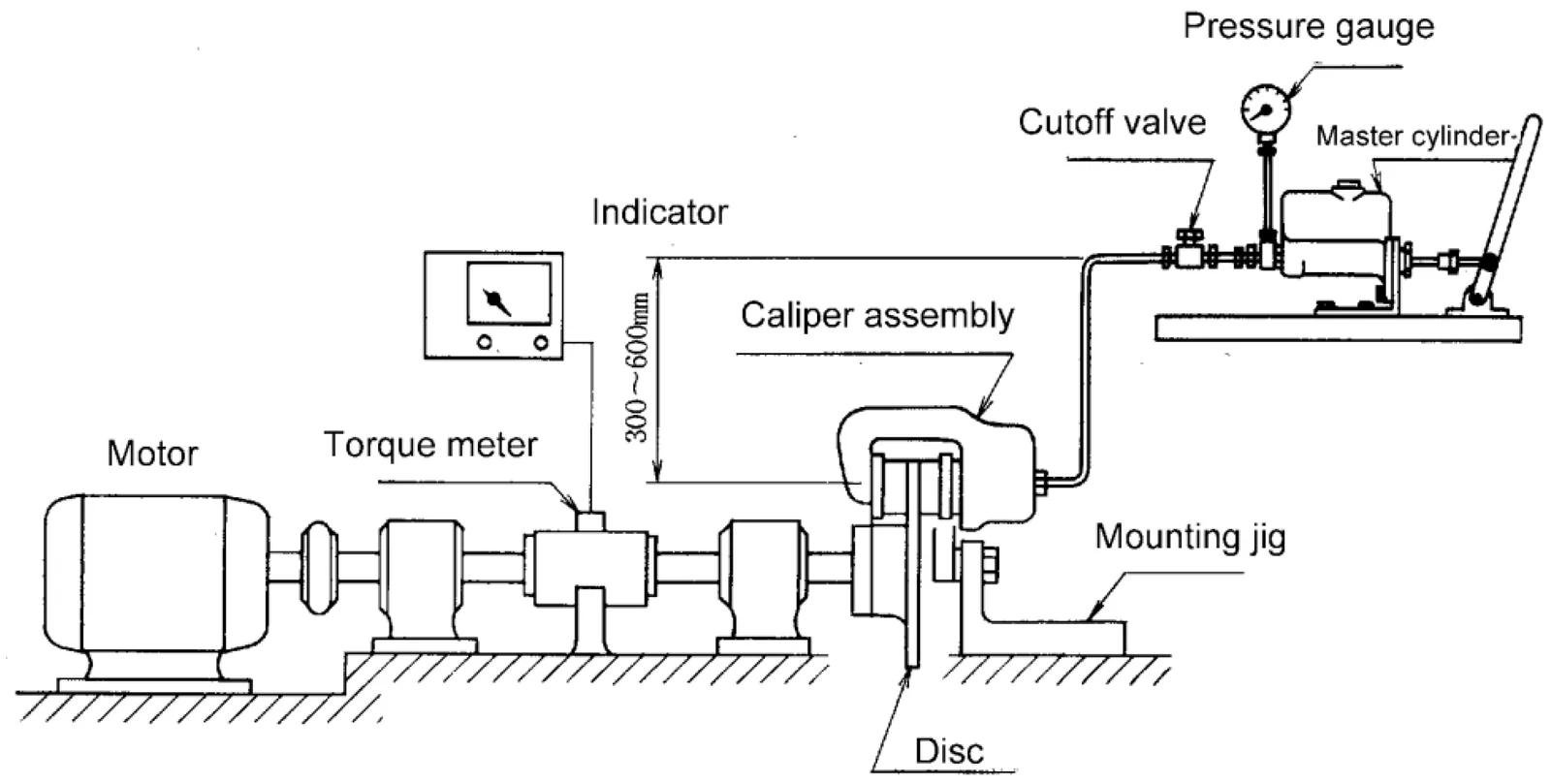
Schematic diagram of the operation of the bench test (friction performance test) device.

In this study, a Link 3000 full-scale inertial test rig was used to conduct characterization tests on braking noise and friction performance, employing C/C-SiC carbon–ceramic brake disks (hereinafter referred to as CCB brake disks) paired with three sets of friction pad combinations. The brake disk had an outer diameter of 312 mm and a thickness of 25 mm. The friction and wear performance of the friction pads was tested under various operating conditions, including braking speed, braking temperature, braking pressure, and braking inertia. Performance characterization was conducted in accordance with the SAE J2522 standard [35], which is widely recognized by global automakers, brake system suppliers, and friction material manufacturers. During the testing process, the inertia test stand incorporated test data for molybdenum disulfide-based, low-metal, copper-free friction pads. To ensure the accuracy of the experimental results, three parallel samples were used for each friction performance testing group to reduce experimental errors. According to the SAE J2522 testing standard, the Link 3000 passenger car brake inertia test bench is used for testing, during which the applied braking pressure is hydraulic or pneumatic pressure applied to the braking system to simulate actual vehicle braking conditions. A schematic diagram of the operation of the bench test (friction performance test) device and test conditions of SAE-J2522 can be seen in Table 3 and Figure 3. Additionally, the equipment can automatically calculate the coefficient of friction (µ) using built-in algorithms combined with the test data. The specific calculation formula is as follows:

$$\mu \;=\;\frac{100\;\times \;Md_{\mathrm{brake}}}{2\;\times \;(P-P_{\mathrm{threshold}})\;\times \;A_{P}\;\times \;r_{\mathrm{eff}}\;\times \;\eta }$$

In the equation: *Md_brake_* is the braking torque, in N·m; *P* is the braking pressure, in MPa; and *P_threshold_* is a constant, with a value of 0.05 MPa; *A_P_* is the area of the brake caliper piston, with a value of 2550 mm^2^; *r_eff_* is the effective braking radius, with a value of 129 mm; and *η* is a constant, with a value of 96%.

### 2.3. Physical Characterization Tests

Analytical techniques such as scanning electron microscopy (SEM), energy-dispersive spectroscopy (EDS), X-ray diffraction (XRD), and energy-dispersive X-ray fluorescence (ED-XRF) were used to conduct a detailed analysis of the microstructure and composition of three types of low-metal, copper-free friction pads containing molybdenum disulfide at different concentrations. A scanning electron microscope was used to examine the surface morphology and microstructure of the low-metal, copper-free friction pads containing molybdenum disulfide. SEM provided high-resolution images for studying the distribution of molybdenum disulfide within the friction pads. Analysis was performed using a JEOL JCM-500 with a magnification range of 10 to 200, while energy-dispersive X-ray spectroscopy (EDS) and energy-dispersive X-ray fluorescence (ED-XRF) were used to quantitatively determine the elemental composition of the friction pads. The phase composition of the friction pads was analyzed using an X-ray diffractometer. XRD analysis helped identify the presence of specific crystalline phases, such as MoS_2_, and measurements were performed using the XSTRESS 3000 portable analyzer. The water porosity (%) and oil porosity (%) of the samples were determined using water and oil (SAE 20), respectively. The water/oil porosity was calculated using the following formula:

$$P\;=\;\frac{w_{f}\;-\;w_{i}}{\mathrm{wi}}$$

*P* = porosity; *w_f_* = weight of the sample after 24 h of immersion; *w_i_* = initial weight of the sample.

For the hydrophobicity test, three samples (A0, A1, and A2) were immersed in deionized water for 24 h, then dried with a hair-dryer, and their weight changes were compared.

To reduce experimental errors, three parallel samples were set for each group in all physical experiments. The types and manufacturer information of the instruments used in the experiment are shown in Table 3.

## 3. Results and Discussion

### 3.1. Microstructural Analysis

**Figure 4:**
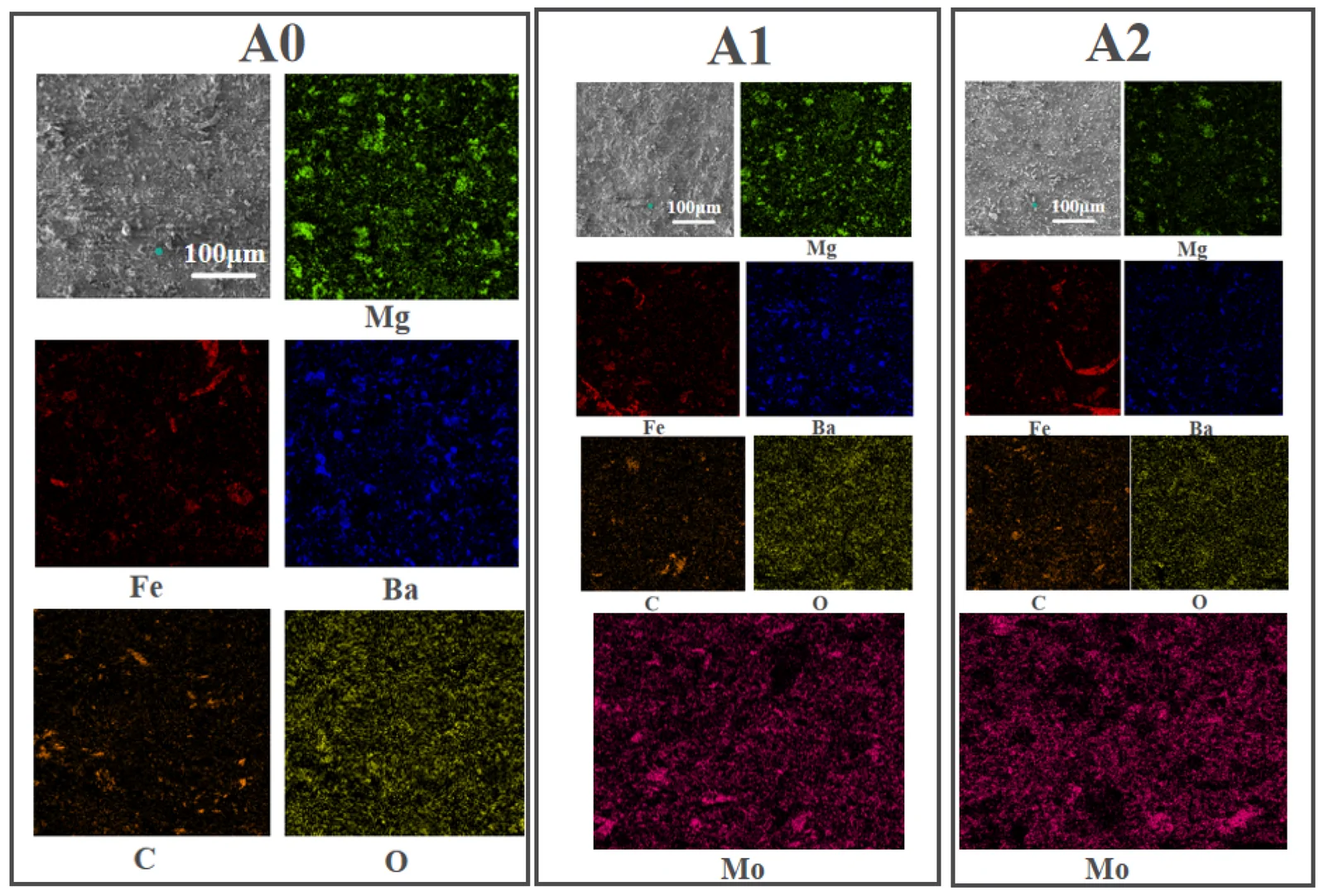
Microstructures of three types of low-metal copper-free molybdenum disulfide friction pads.

**Figure 5:**
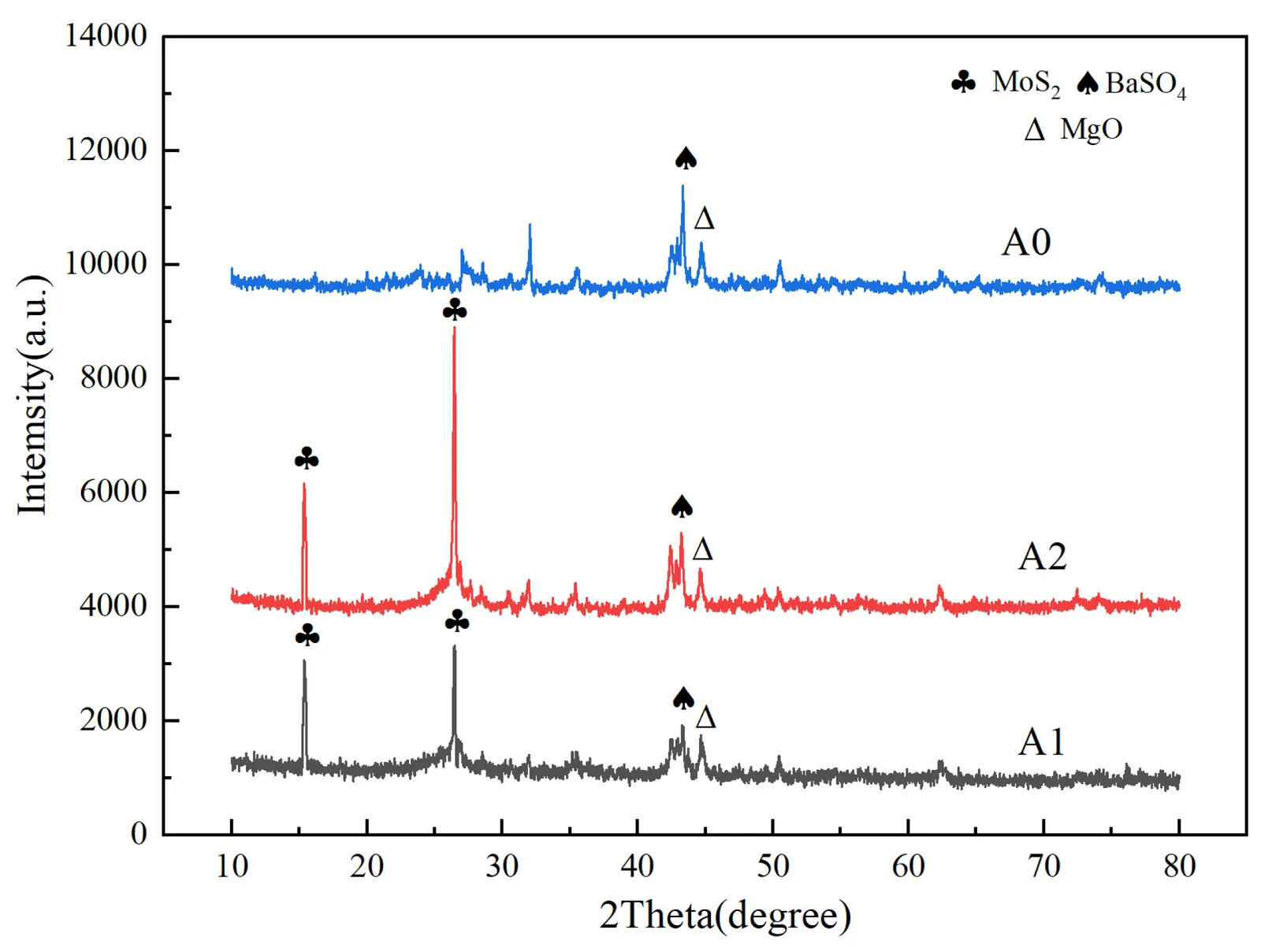
XRD patterns of three types of low-metal copper-free molybdenum disulfide friction pads.

As shown in Table 1, the friction material prepared in this study is a copper-free system, which complies with current environmental regulations. On 21 January 2015, environmental protection agencies and the automotive industry signed an agreement to regulate the copper content in automotive brake pads; this standard has now been fully implemented across the braking industry. Figure 4 and Figure 5, together with Table 4, present the microstructural morphology (SEM), EDX, XRD, and XRF elemental distribution characteristics of three low-metal, copper-free friction pads with different molybdenum disulfide (MoS_2_) contents, providing important reference data for the mechanistic study of friction materials. The material is primarily composed of elements such as iron, barium, calcium, molybdenum, sulfur, carbon, oxygen, and silicon. The synergistic interaction of these components endows the material with excellent friction and wear resistance properties. Iron represents the steel fibers, which are used to enhance the strength of the friction pad matrix; silicon carbide (SiC), composed of silicon and carbon, acts as a friction enhancer to increase the coefficient of friction; and barium compounds serve as friction-modifying additives, providing both friction-reducing and wear-resistant effects. As observed in Figure 4, the solid lubricant MoS_2_ is uniformly distributed in the A1 and A2 specimens, with no agglomeration; in the A0 specimen, which did not contain MoS_2_, EDS analysis detected no corresponding elements.

### 3.2. Physical Properties

As shown in Table 5, the densities of the A0, A1, and A2 specimens, as measured using a density tester, were 3.21; 3.28; and 3.36 g/cm^3^, respectively. Their shear strengths at room temperature were 5.60 ± 0.50 MPa, 6.09 ± 0.41 MPa, and 6.69 ± 0.52 MPa, respectively; their water-soaked porosity values were 15.50 ± 0.04%, 14.77 ± 0.04%, and 12.73 ± 0.04%, respectively, with porosity ranked as A0 > A1 > A2. Table 6 shows that sample A2 absorbed the least water in weight after 24 h of soaking in deionized water, demonstrating the best hydrophobic performance. Overall, the A2 specimen combines higher density and shear strength, lower porosity, and the strongest hydrophobic performance. A comparison of the results shows that the A2 specimen performs best in terms of physical and mechanical properties such as density and shear strength, while the performance of the A1 and A0 specimens decreases in that order. Additionally, the water porosity of both A1 and A2 is lower than that of A0, a pattern that is generally consistent with commercial friction materials. MoS_2_ particles are characterized by small particle size and high specific surface area. Compared to other lubricating fillers, such as graphite, they can form more significant interfacial bonding effects within the friction material, resulting in a more compact internal structure and further enhanced overall stability. As shown in Table 6, the A1 and A2 specimens exhibited significantly better hydrophobicity than the matrix A0 due to the introduction of the solid lubricant MoS_2_. MoS_2_ has a typical layered structure composed of alternating layers of molybdenum and sulfur atoms. Molybdenum atoms possess unfilled d-orbitals that can interact strongly with sulfur atoms, forming stable covalent bonds within the layers. This structure imparts a smooth surface morphology to the material, endowing it with excellent resistance to water wetting. In composite friction materials, MoS_2_ forms a hydrophobic barrier on the surface layer, effectively enhancing the material’s overall performance under humid operating conditions. The higher contact angles observed in wetting tests also confirm that water has difficulty wetting and affecting this type of friction material [36].

**Figure 6:**
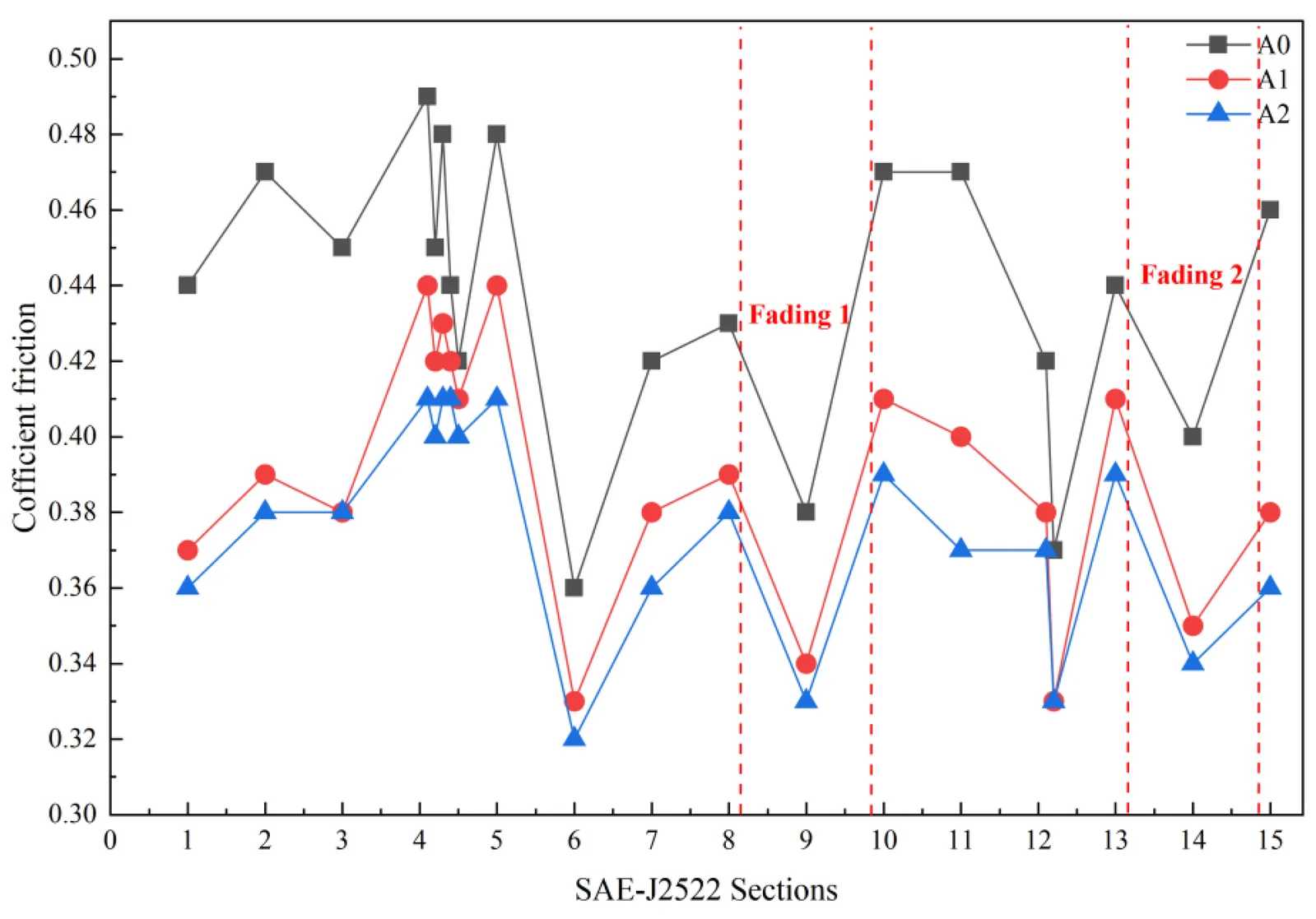
Friction coefficients of three types of low-metal copper-free molybdenum disulfide friction pads.

**Figure 7:**
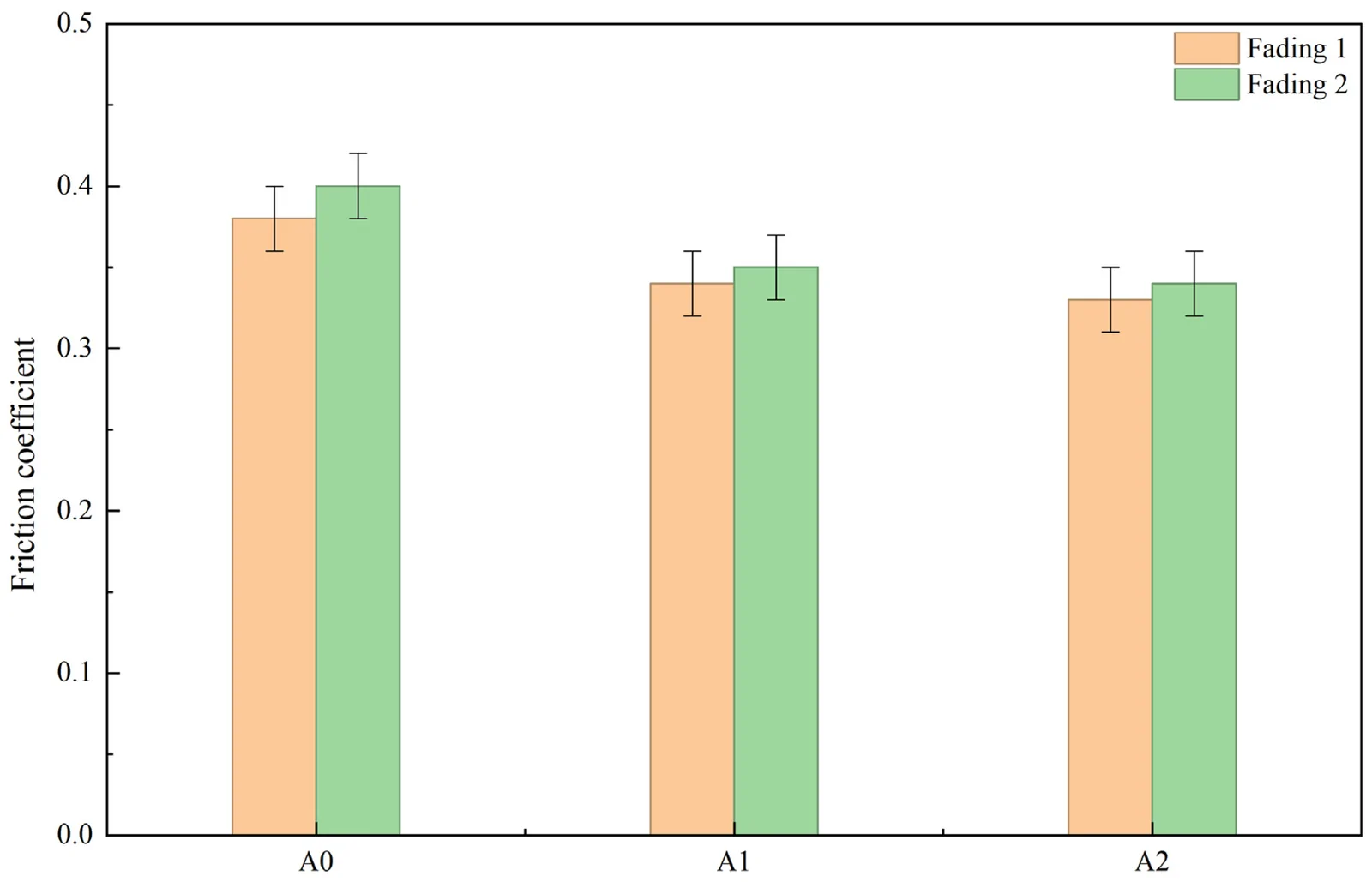
Fading coefficients of three types of low-metal, copper-free molybdenum disulfide friction pads.

**Figure 8:**
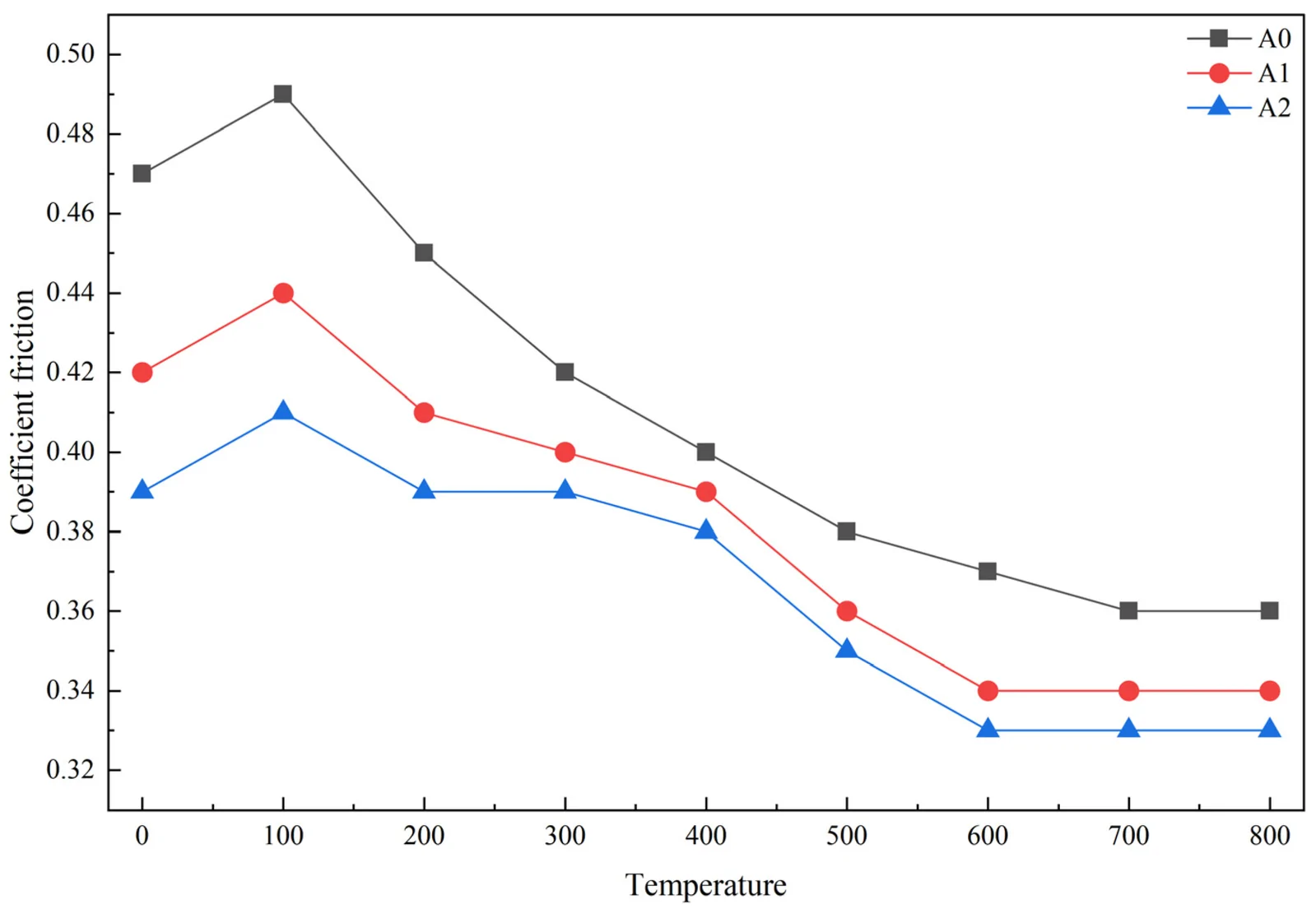
Friction coefficient distribution of three types of low-metal, copper-free molybdenum disulfide friction pads.

The friction coefficient curves and test data for the A0, A1, and A2 friction pads are shown in Figure 6 and Figure 7 and Figure 8 and Table 7, Table 8 and Table 9, respectively. The test results show that the average friction coefficient of the A0 sample reached 0.437, which was the highest value among all test groups; the A2 sample had the lowest average friction coefficient at 0.375, while the A1 sample had an average friction coefficient of 0.390. Table 8 shows the friction coefficients for all operating conditions, A0, A1, and A2. Table 9 shows a trend consistent with that in Table 8: the A3 friction pad exhibited the least mass loss, and when paired with a CCB brake disk, this sample also had the lowest system wear rate. In summary, the MoS_2_-doped A1 and A2 friction materials demonstrate good composite lubrication effects, with wear rates significantly lower than those of the MoS_2_-free base material A0. Figure 6 shows the friction coefficient variation curves for different specimens; the overall trends of the curves across the groups are similar. Compared to the A0 and A1 specimens, the A2 specimen exhibits smaller fluctuations in friction coefficient and a more stable friction state. The results of the pin-on-disk friction test indicate that the specific wear rates of the three groups of specimens, from lowest to highest, are A2, A1, and A0. As a solid lubricant, MoS_2_ forms a lubricating film on the friction contact surface, thereby reducing interfacial friction and minimizing material loss. Its lubrication mechanism essentially stems from its layered crystal structure and low interlayer bonding energy: weak interlayer forces cause the crystal layers to undergo shear dislocation very easily during relative sliding, forming a uniform and dense lubricating layer on the friction surface that significantly reduces the coefficient of friction. This interfacial film not only reduces friction but also prevents direct contact with the substrate, thereby providing effective protection for the friction surfaces [6]. MoS_2_ combines a low coefficient of friction with excellent thermal stability and outstanding self-lubricating properties. It maintains stable lubrication capabilities even under severe operating conditions, making it suitable for a wide range of complex operating conditions and conferring high practical application value.

The research results of Sheikh et al. [18] indicate that molybdenum disulfide can exert a certain lubricating effect in the range of low temperature to 800 °C and Gao et al. [9] pointed out that when the temperature exceeds 350 °C, the molybdenum disulfide film will lose a large number of atomic layers and undergo severe oxidation, and its high-temperature lubrication effect will begin to be significantly reflected. This is consistent with the results in Figure 8, where the friction coefficient rapidly decreases in the range of 350–600 °C. The temperature tends to stabilize within the range of 600–800 °C, as the lubricant in the friction plate has been depleted in the high-temperature environment and can no longer provide lubrication.

As shown in Table 6 and Table 9, A2, which has a lower CoF value, results in less wear on the friction pad and brake disk during braking. This is because MoS_2_ has a more regular structure and stronger interlayer bonding, leading to more consistent and smooth friction behavior, which in turn results in less heat generation and reduced wear between the friction pad and the friction pair [36].

**Figure 9:**
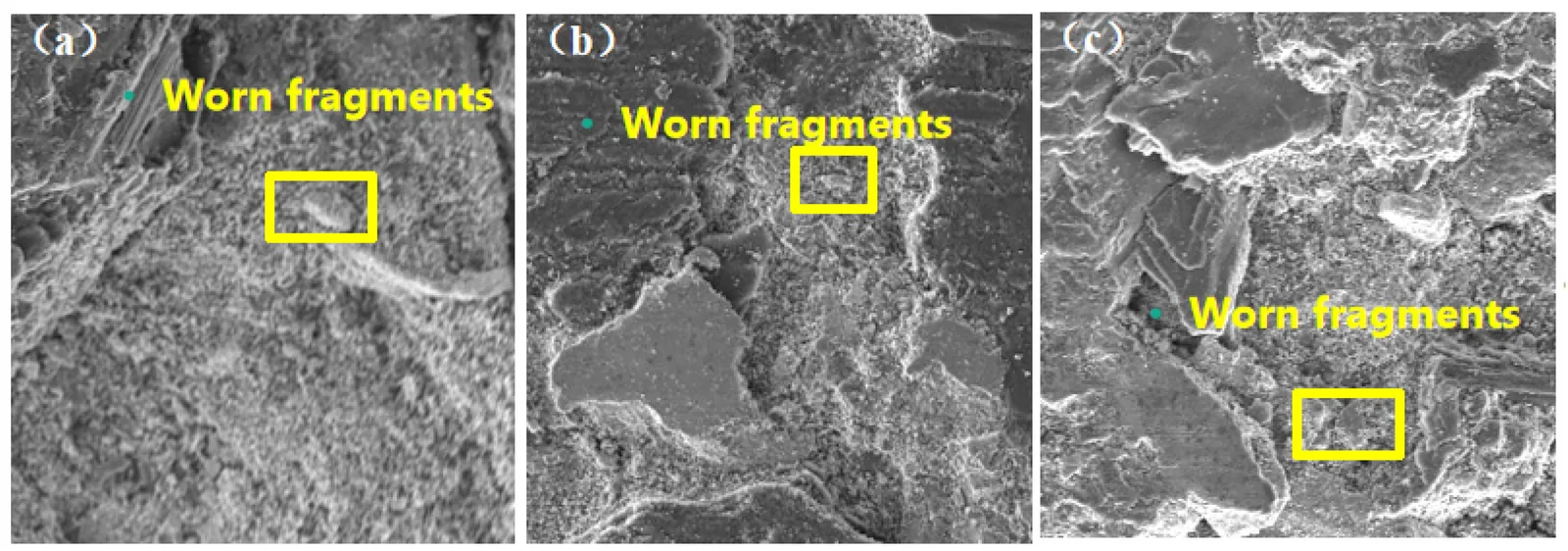
SEM image showing a close-up of the worn surface of a friction pad; (**a**) A0 sample, (**b**) A1 sample, (**c**) A2 sample.

As shown in Figure 9, the amount of abrasive particles in Figure 9a is significantly higher than in Figure 9b,c. The high temperatures under braking conditions can cause the solid lubricant MoS_2_ to form a protective film at the friction interface, effectively mitigating friction pad wear. Plowing tends to cause delamination between fibers and the matrix, inducing surface fatigue cracks, and is the primary cause of severe abrasive wear in friction materials. Abrasive wear refers to the process in which two contacting surfaces undergo intense friction and the peeling off of materials with low wear resistance, whereas the adhesion and transfer of surface particles correspond to adhesive wear [17]. The primary and secondary friction platforms are the two key structures on the friction surface of brake pads during braking. The primary friction platform is the contact area that forms first on the friction surface; as the main load-bearing surface, it is the foundation for the stable service life of the friction material. As wear debris continuously accumulates around the primary friction platform, a secondary friction platform gradually forms. As shown in Figure 9a, the A0 specimen without MoS_2_ exhibits severe wear of the primary friction platform, with a large number of abrasive particles forming on its surface. In contrast, as seen in Figure 9b,c, the A1 and A2 specimens doped with MoS_2_ exhibit a more intact primary friction platform structure, with a significantly reduced number of abrasive particles. This is because MoS_2_ has a layered structure that acts as a third body for sliding and rolling at the friction interface. The thin, flake-like particles it forms can fully penetrate the contact interface, providing a lubricating effect and mitigating hard contact between the friction surfaces. At the same time, the sliding and rolling effects of these particles effectively suppress surface plowing behavior, significantly reducing abrasive wear [37,38].

The formation mechanism of abrasive wear in this study is as follows: during braking, fractured SiC particles on the brake disk surface and hard phases in the friction material act as abrasive particles at the friction interface, leading to abrasive wear. As shown in Figure 9, steel fibers in the friction material undergo plastic deformation during braking, resulting in the continuous accumulation and compaction of wear debris on the surface. After adding molybdenum disulfide (MoS2), Mo elements are uniformly distributed across the entire friction surface. As a solid lubricant, MoS_2_ spreads evenly on the friction surface, forming a complete and smooth friction film, which helps reduce the friction coefficient of the brake pair and minimize wear on the friction material. Consequently, the A0 sample without MoS2 exhibits the highest amount of wear debris on its surface [39].

**Figure 10:**
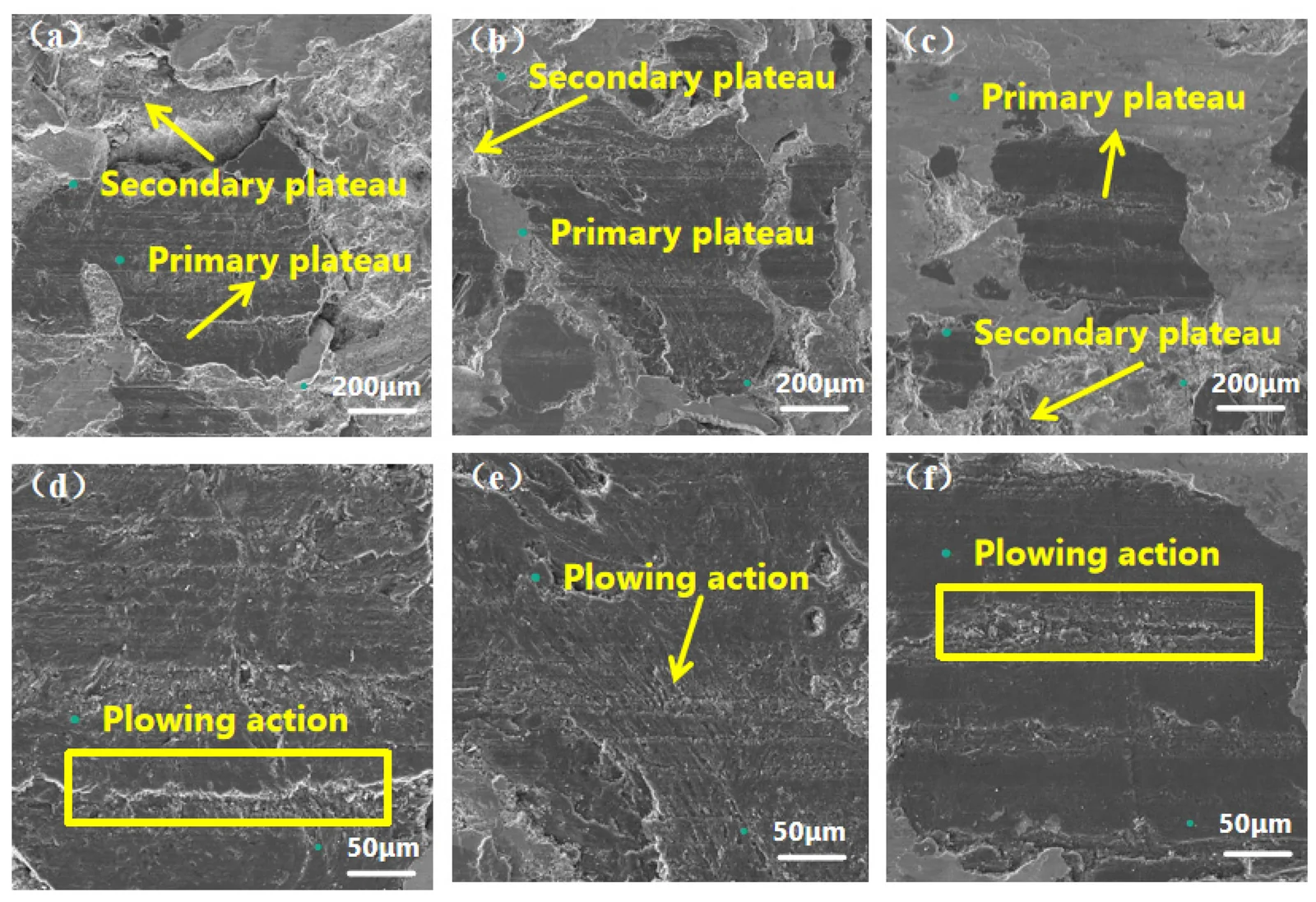
BSE-SEM micrographs of the worn surface of (**a**,**d**) A0 and (**b**,**e**) A1 and (**c**,**f**) A3.

Analysis Based on Figure 10: Abrasive wear was the predominant mechanism at the interface between the A0 specimen and its counterpart; the wear mechanisms of the A1 and A2 specimens and the CCB brake disk were essentially the same. An increase in MoS_2_ content effectively reduced the wear volume of both types of specimens, as shown in Figure 10e,f, respectively. Figure 10d–f show the surface wear characteristics of the three groups of friction pads paired with CCB as friction pads, respectively. Observation of Figure 10f reveals that the CCB paired with the A2 specimen exhibits the fewest plow marks and the shallowest depth. This is attributed to the fact that MoS_2_ possesses a high specific energy and wear can interact with the iron matrix to form an additional stable protective layer on the friction contact surface, thereby achieving a good wear-reducing effect [1].

As shown in Figure 10, the A0 sample (without MoS_2_) exhibits a high concentration of surface wear particles and severe damage on the friction plateau, indicating severe adhesive wear and abrasive wear. In contrast, the A1 and A2 samples display more intact surfaces with less wear debris, which is a direct result of MoS_2_ inhibiting adhesive wear. The MoS_2_ layer consists of strong covalent bonds within the layers, while the interlayer bonding relies solely on weak van der Waals forces, making lateral sliding highly prone to occur. The interlayer shear strength is only about 1–2 MPa, causing interfacial shear to predominantly occur within the MoS_2_ layer rather than in the base material, significantly weakening the driving force for adhesive junction formation. During sliding, MoS_2_ forms a transfer film on the counter surface, with the (0001) basal plane oriented along the sliding direction. This transfer film separates the two friction surfaces, effectively preventing direct metal-to-metal (friction pad–brake disk) contact and physically inhibiting large-scale cold welding and material transfer. Additionally, MoS_2_ flakes in the wear debris act as a third body at the interface, bearing loads through sliding and rolling (shear stress can also induce the curling of MoS_2_ nanosheets into nanospheres, generating a rolling effect), transforming adhesive detachment from “pulling” to “rolling” and reducing plowing effects and material tearing. In summary, these characteristics enable the A1 and A2 samples with MoS_2_ additives to exhibit significantly less adhesive wear and abrasive wear compared to the A0 sample [40].

**Figure 11:**
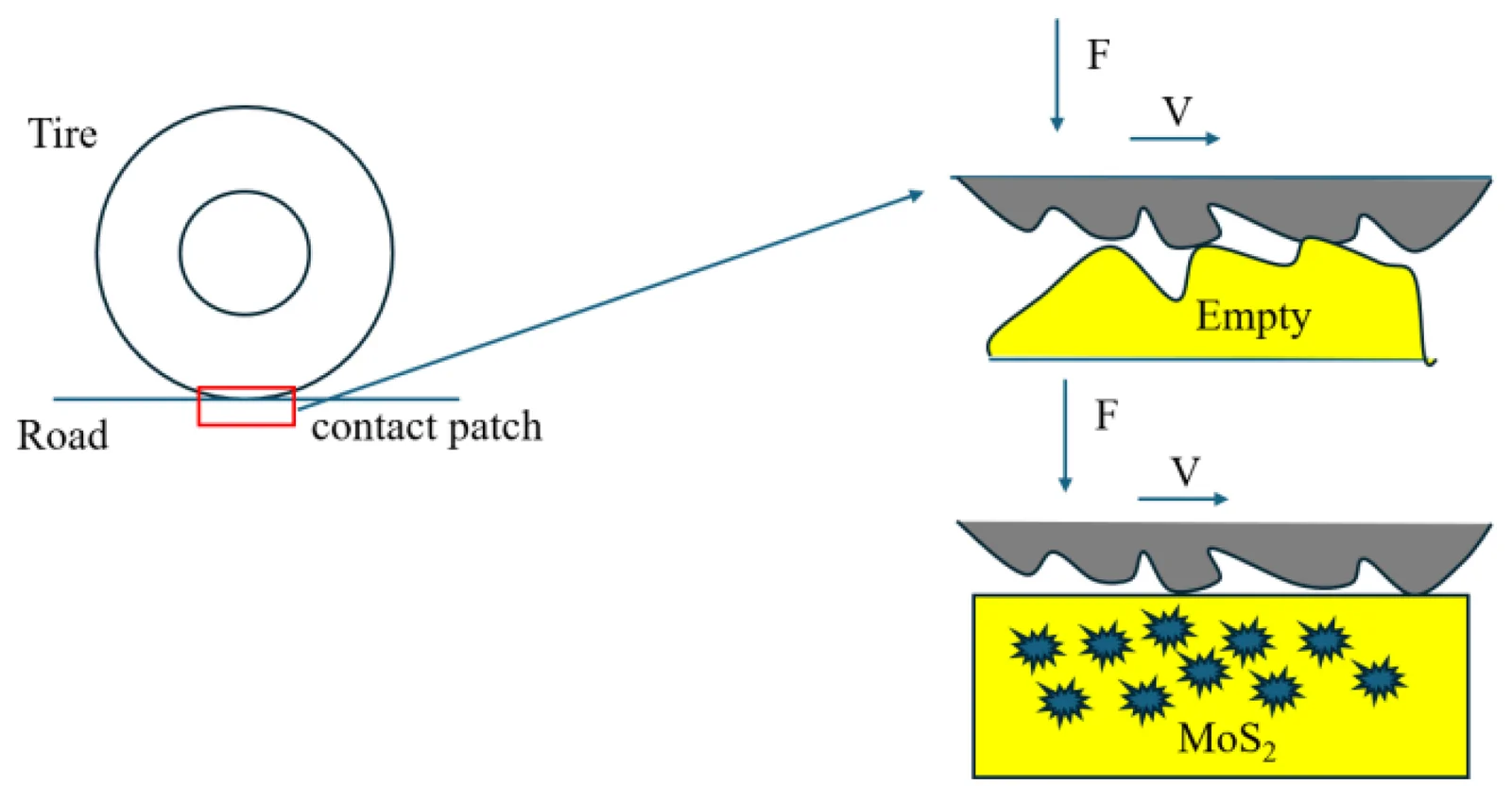
Mechanism by which molybdenum disulfide reduces wear.

Figure 11 visually illustrates the mechanism by which MoS_2_ modulates the coefficient of friction. Due to its tiny size, few-layer MoS_2_ can fill the micropores and interfacial defects within low-metal friction composites, promoting densification of the material matrix, effectively suppressing adhesive wear, and enhancing wear resistance. The stress concentration mitigation effect resulting from the filling of material pores, combined with the lubricating effect of interlayer slippage in MoS_2_ that dissipates interfacial shear energy, creates a synergistic effect that serves as the core mechanism for enhancing the overall performance of friction materials.

## 4. Conclusions

This article systematically analyzes the friction pair composed of low-metal friction pads containing molybdenum disulfide (MoS_2_) and CCB (carbon ceramic brake disk), and draws the following main conclusions:

1. MoS_2_ has a typical layered structure, consisting of alternating layers of molybdenum and sulfur atoms; molybdenum atoms contain unfilled d orbitals, which can strongly interact with sulfur atoms and form stable covalent bonds within the layer. This structure endows the material with a smooth surface morphology, significantly improving the hydrophobic performance of the brake pad. Therefore, A2 brake pads with the highest MoS_2_ content exhibit the best hydrophobic performance.
2. The layered crystal structure and low interlayer bonding energy of MoS_2_ are the fundamental reasons for its excellent lubrication performance: the weak interlayer forces make the crystal layers prone to shear dislocations during relative sliding, and form a uniform and dense lubrication layer on the friction surface, resulting in a lower coefficient of friction (CoF) and specific wear rate of the brake pad. Therefore, A2 brake pads have minimal wear on both brake pads and brake disks during the braking process.
3. MoS_2_ has a high specific energy and can interact with the iron matrix to form an additional stable protective layer on the friction contact surface, thereby achieving a good wear reduction effect, effectively reducing plowing effect and lowering wear.

## Figures and Tables

**Table 1:** Approximate composition of three types of low-metal friction materials containing MoS_2_ (Wt.%).

Raw Materials (wt%)	A0	A1	A2
Powder resin	10%	10%	10%
Rubber powder	2%	2%	2%
Steel fiber	30%	30%	30%
Aramid fiber	2%	2%	2%
Mineral fiber	5%	5%	5%
MgO	5%	5%	5%
MoS_2_	0%	4%	8%
Artificial graphite	10%	10%	10%
BaSO_4_	28%	24%	20%
ZrSO_4_	8%	8%	8%

**Table 2:** Test conditions of SAE-J2522.

Section	Test Name	Condition
1	Green μ characteristic	80 → 30 km/h; 3.0 MPa; 30 stops
2	Burnish	80 → 30 km/h; 1.0–5.0 MPa; 64 stops
3	Characteristic value 1	80 → 30 km/h; 3.0 MPa; 6 stops
4.1	Speed/pressure sensitivity	40 → 5 km/h; 1.0–5.0 MPa; 5 stops
4.2		80 → 40 km/h; 1.0–5.0 MPa; 5 stops
4.3		120 → 80 km/h; 1.0–5.0 MPa; 5 stops
4.4		160–130 km/h; 1.0–5.0 MPa; 5 stops
4.5		180 → 150 km/h; 1.0–5.0 MPa; 5 stops
5	Characteristic value 2	80 → 30 km/h; 3.0 MPa; 6 stops
6	Cold braking	100 → 5 km/h; 160 → 10 km/h
7	Motorway braking	100 → 5 km/h; 160 → 10 km/h; 60% deceleration; 2 stops
8	Characteristic value 3	80 → 30 km/h; 3.0 MPa; 18 stops
9	1st fading	100 → 5 km/h; 40% deceleration; 15 stops
10	Recovery 1	80 → 30 km/h; 3.0 MPa; 18 stops
11	Temperature/pressure sensitivity 100 °C	80 → 30 km/h; 1.0–5.0 MPa; 5 stops
12.1	Temperature/pressure sensitivity 500 °C	80 → 30 km/h; 1.0–5.0 MPa; 9 stops
12.2		80 → 30 km/h; 1.0–5.0 MPa; 5 stops
13	Recovery 2	80 → 30 km/h; 3.0 MPa; 18 stops
14	2nd fading	100 → 5 km/h; 40% deceleration; 15 stops
15	Recovery 3	80 → 30 km/h; 3.0 MPa; 18 stops

**Table 3:** Main experimental instruments and equipment.

Equipment Type	Model Number	Manufacturer
Bench testing machine	Link 3000	Link (USA Plymouth, MI)
Scanning electron microscopy (SEM)	JCM-500	JEOL (Japan)
X-ray diffraction (XRD)	XSTRESS 3000	IDONAMO (China)
Energy-dispersive X-ray fluorescence (XRF)	ARL QUANT’X EDXRF	Thermo Fisher (USA)
Shear testing machine	MTS810	Shenzhen Sansheng Zongheng Technology Co., Ltd. (China)

**Table 4:** Elements detected by XRF in three types of low-metal, copper-free molybdenum disulfide friction pads.

Samples	Fe (%)	Ba (%)	Mg (%)	C (%)	O (%)	Mo (%)
A0	29.33	28.20	4.88	1.27	6.33	/
A1	29.45	23.79	5.03	1.34	6.18	3.96
A2	29.62	20.11	4.97	1.29	6.62	8.01

**Table 5:** Physical and chemical properties of the three types of friction pads.

Samples	Density (g/cm^3^)	Sheer Strength (MPa)	Compressive Strength (MPa)	Water Porosity (%)
A0	3.21 ± 0.03	5.60 ± 0.50	51.30 ± 1.88	15.50 ± 0.06
A1	3.28 ± 0.02	6.09 ± 0.41	57.52 ± 1.31	14.77 ± 0.04
A2	3.36 ± 0.03	6.69 ± 0.52	61.77 ± 1.56	12.73 ± 0.02

**Table 6:** Weight Results for the Three Types of Friction Pads After 24-Hour Soaking in Deionized Water.

Samples	Weight Pad (g) Before	Weight Pad (g) After	Weight Pad (g) Loss
A0	659.69	662.29	2.60
A1	666.01	667.76	1.75
A2	659.28	660.22	0.94

**Table 7:** Statistics on the coefficients of friction for three types of friction pads.

SAE-J2522 Section	μ A0	μ A1	μ A2
1	0.44	0.37	0.36
2	0.47	0.39	0.38
3	0.45	0.38	0.38
4.1	0.49	0.44	0.41
4.2	0.45	0.42	0.4
4.3	0.48	0.43	0.41
4.4	0.44	0.42	0.41
4.5	0.42	0.41	0.4
5	0.48	0.44	0.41
6	0.36	0.33	0.32
7	0.42	0.38	0.36
8	0.43	0.39	0.38
9	0.38	0.34	0.33
10	0.47	0.41	0.39
11	0.47	0.4	0.37
12.1	0.42	0.38	0.37
12.2	0.37	0.33	0.33
13	0.44	0.41	0.39
14	0.4	0.35	0.34
15	0.46	0.38	0.36

**Table 8:** Statistics on the average and maximum coefficients of friction for the three types of friction pads.

Samples	μmax	μavg
A0	0.49	0.437
A1	0.44	0.390
A2	0.41	0.375

**Table 9:** Mass loss of friction pads and friction pads.

Samples	Weight Pad (g) Before	Weight Pad (g) After	Weight Pad (g) Loss	Percentage Mass Loss (%)	Weight Disk (g) Before	Weight Disk (g) After	Weight Disc (g) Loss	Percentage Mass Loss (%)
A0	468.6	453.1	15.5	3.30	4138.6	4137.0	1.6	0.04
A1	531.8	520.4	11.4	2.14	4290.3	4289.1	1.2	0.03
A2	519.7	510.9	8.8	1.69	4266.7	4265.9	0.8	0.02

## Data Availability

The original contributions presented in this study are included in the article. Further inquiries can be directed to the corresponding author.
